# Low Muscle Mass, Sleep Quality, and Quality of Life Among Older Chinese Adults: A Cross-Sectional Study

**DOI:** 10.3390/healthcare14213762

**Published:** 2026-11-04

**Authors:** Yanan Lin, Xiaorong Lin, Rubin Chen, Sengbin Yang, Ying Xiong, Wenwen Cao, Jianyu Que, Haibin Zhang

**Affiliations:** Xiamen Xianyue Hospital, Xianyue Hospital Affiliated with Xiamen Medical College, Fujian Psychiatric Center, Fujian Clinical Research Center for Mental Disorders, Xiamen 361000, China

**Keywords:** low muscle mass, sleep quality, quality of life, older adults, China, Chinese Longitudinal Healthy Longevity Survey

## Abstract

**Background:** Low muscle mass (LMM), poor sleep quality, and reduced quality of life (QOL) are common concerns in older adults. This study examined their cross-sectional associations and explored the statistical indirect association involving sleep quality, with particular attention to sensitivity to body-size adjustment and alternative model specifications. **Methods:** We analyzed data from 9366 adults aged 65 years or older in the 2018 Chinese Longitudinal Healthy Longevity Survey. LMM was classified using anthropometrically predicted appendicular skeletal muscle mass, while sleep quality and overall QOL were assessed using single-item self-reports. Regression-based indirect associations were estimated with 5000 bootstrap resamples. Sensitivity analyses examined alternative measurements, body-size adjustment, depressive symptoms, model ordering, missing-data assumptions, and sampling weights. **Results:** Anthropometrically estimated LMM was associated with poorer sleep quality (B = −0.066, 95% CI: −0.110 to −0.022) and lower QOL (total association: B = −0.073, 95% CI: −0.108 to −0.039). Better sleep quality was associated with higher QOL (B = 0.163, 95% CI: 0.147 to 0.178). The indirect association was small (B = −0.011, 95% bootstrap CI: −0.018 to −0.003) and attenuated after continuous body mass index adjustment (B = −0.001, 95% bootstrap CI: −0.010 to 0.008). Adjustment for depressive symptoms further attenuated the estimated indirect association, while a reverse-order model also yielded a non-zero indirect association (B = −0.020). Residual-confounding sensitivity analyses indicated dependence on assumptions about unmeasured common causes. **Conclusions:** Anthropometrically estimated LMM was associated with poorer perceived sleep quality and overall QOL, but the estimated indirect association involving sleep quality was small and sensitive to body-size adjustment, psychological covariates, and model specification. These cross-sectional findings do not establish a muscle-specific association independent of overall body size or a causal sleep-mediated pathway. Longitudinal studies incorporating directly measured body composition and validated multidimensional sleep and QOL assessments are needed to clarify these relationships.

## 1. Introduction

Sarcopenia is an age-related skeletal muscle disorder characterized by reductions in muscle mass, strength, and physical performance and is associated with disability, falls, frailty, mortality, and substantial healthcare burden in older populations [1,2]. Low muscle mass (LMM), a central component of sarcopenia assessment [3], has also been associated with poorer quality of life (QOL) [4,5,6]. Maintaining functional ability and perceived well-being is a central objective of healthy ageing [7]. Understanding how indicators of muscle health relate to sleep and subjective QOL may therefore contribute to a broader description of health status in older populations.

Sleep quality is another important dimension of health and well-being in later life. Conceptual models of health-related QOL, including those proposed by Wilson and Cleary and subsequently refined by Ferrans et al., describe relationships among physiological status, symptoms, functional health, and subjective evaluations of well-being [8,9]. These frameworks provide a broad rationale for examining muscle-related indicators, sleep quality, and QOL together, but they do not establish a specific temporal sequence among the measures used in the present study. Previous research has reported associations between muscle-related characteristics and sleep outcomes [10,11,12], as well as between sleep quality and QOL in older adults [13,14].

The relationships among muscle health, sleep, and perceived QOL may be reciprocal and influenced by shared determinants. Longitudinal studies have suggested that sleep characteristics may precede changes in muscle-related outcomes [15], while sleep quality and subjective well-being may influence each other over time [16]. Methodological research has also shown that cross-sectional mediation analyses may not reproduce longitudinal processes when temporal ordering is unknown [17,18].

Studies using the 2018 Chinese Longitudinal Healthy Longevity Survey (CLHLS) have reported associations of anthropometrically estimated LMM with depressive symptoms and subjective well-being, as well as relationships among sleep quality and psychosocial characteristics [19,20,21]. However, the concurrent relationships among estimated LMM, sleep quality, and global QOL have not been fully characterized within a common analytical framework that explicitly examines their sensitivity to body size, psychological symptoms, and alternative model specifications. Such an analysis may help clarify the descriptive value and limitations of these routinely available survey measures, without assuming that they identify a muscle-specific or causal pathway.

Potential heterogeneity was examined as a secondary question. Ageing-related changes in body composition differ between men and women [22], and sex differences have also been reported in sleep characteristics [23]. Marital relationships and social support may influence sleep through shared routines, caregiving, relationship quality, and the home sleep environment [24,25]. Sex and marital status were therefore examined as exploratory sources of heterogeneity in the regression-based associations, without presuming that subgroup differences would identify distinct causal mechanisms.

Accordingly, this study used data from the 2018 CLHLS to examine the concurrent associations among anthropometrically estimated LMM, self-reported sleep quality, and overall QOL in Chinese adults aged ≥65 years. A regression-based indirect-association model was used as an exploratory statistical decomposition rather than a test of causal mediation. We further evaluated the sensitivity of these associations to body-size adjustment, depressive symptoms, alternative exposure and outcome definitions, model ordering, and missing-data assumptions. Exploratory analyses by sex and marital status were conducted as secondary objectives.

## 2. Materials and Methods

### 2.1. Study Design and Participants

We conducted a cross-sectional analysis using data from the 2018 wave of the Chinese Longitudinal Healthy Longevity Survey (CLHLS), a population-based survey of older adults conducted across 22 provinces in China. The survey used a multistage sampling design and collected demographic, socioeconomic, health, behavioral, and anthropometric information through structured household interviews and physical measurements [26].

The CLHLS protocol was approved by the Biomedical Ethics Committee of Peking University (IRB00001052-13074). Written informed consent was obtained from participants or their legally authorized representatives during the original CLHLS data collection. The 2018 wave was selected because it provided the complete set of anthropometric measurements, sleep-quality data, QOL items, and covariates required for the present analysis.

**Figure 1:**
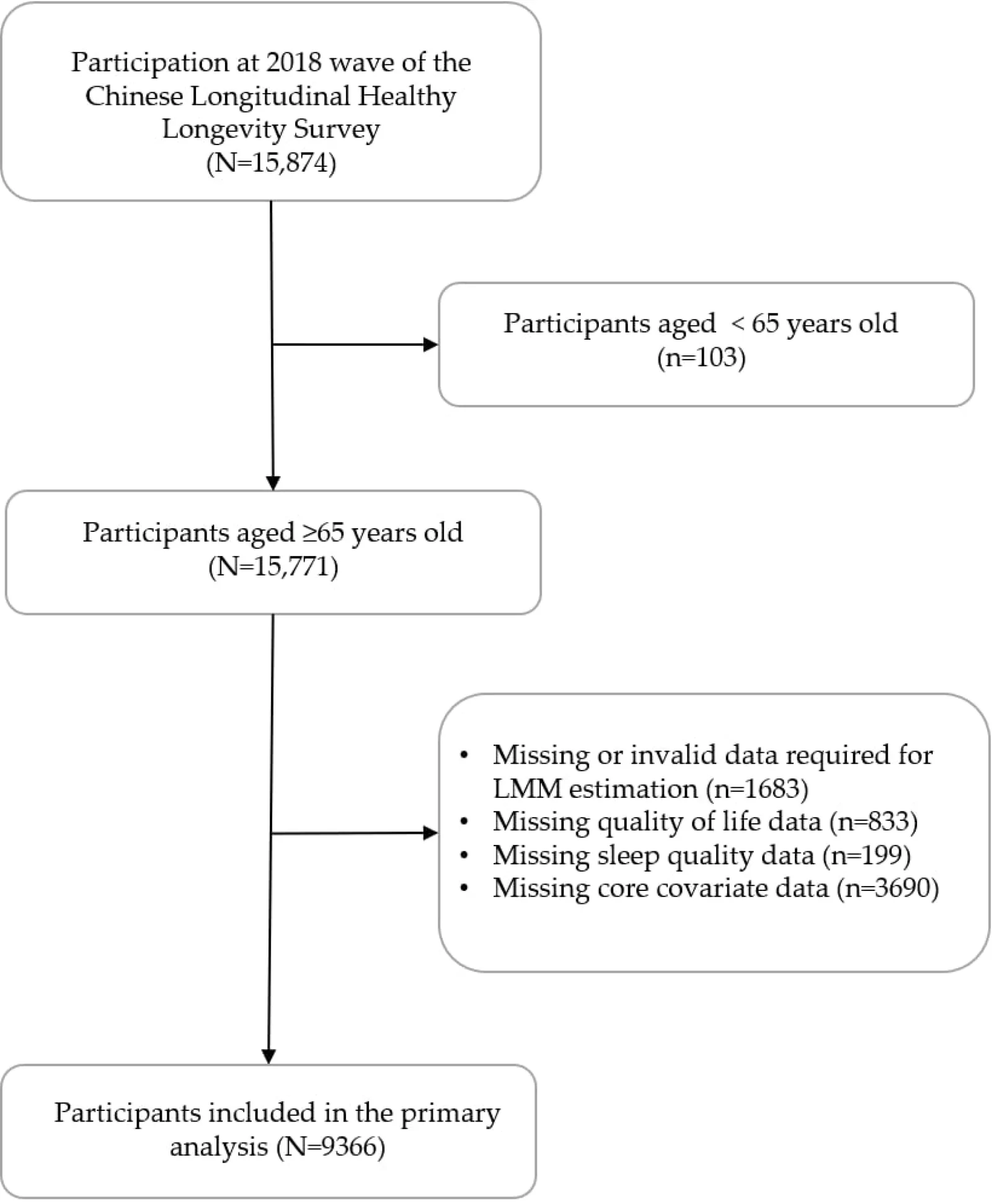
Flow diagram of participant selection for the primary analysis.

The 2018 dataset included 15,874 participants. We excluded 103 participants aged <65 years, leaving 15,771 age-eligible participants. Participants were then sequentially excluded because of missing or invalid anthropometric information required to estimate low muscle mass (*n* = 1683), missing overall quality-of-life data (*n* = 833), missing sleep-quality data (*n* = 199), or missing data on at least one prespecified core covariate (*n* = 3690). The final primary complete-case sample comprised 9366 participants. Activities of daily living (ADL) impairment was not required for inclusion because it was not part of the primary adjustment set. Participant selection is shown in Figure 1.

The primary analysis was restricted to participants with complete data for the anthropometrically derived exposure, sleep quality, overall QOL, and prespecified core covariates. Consequently, the complete-case sample may differ systematically from the full age-eligible population. The extent and potential implications of this selection were examined in Supplementary missing-data analyses (Supplementary Tables S6 and S7).

### 2.2. Measures

#### 2.2.1. Anthropometrically Estimated Low Muscle Mass

Direct body-composition measurements using dual-energy X-ray absorptiometry (DXA) or bioelectrical impedance analysis were not available in the 2018 CLHLS. Appendicular skeletal muscle mass (ASM) was therefore estimated using the anthropometric equation developed and validated by Kawakami et al. [27]:

ASM (kg) = 2.955 × sex (male = 1, female = 0) + 0.255 × weight (kg) − 0.130 × waist circumference (cm) + 0.308 × calf circumference (cm) + 0.081 × height (cm) − 11.897

Appendicular skeletal muscle mass index (ASMI) was calculated as estimated ASM divided by height squared (kg/m^2^). For the primary analysis, anthropometrically estimated LMM was operationally defined as ASMI <7.0 kg/m^2^ in men and <5.7 kg/m^2^ in women. These thresholds correspond to the AWGS 2019 ASMI cutoffs commonly applied to bioelectrical impedance measurements [3] and were retained to facilitate comparability with previous CLHLS studies using anthropometrically estimated muscle mass [28,29]. These cutoffs were selected to facilitate comparability with previous studies, rather than because their diagnostic accuracy had been established for anthropometrically predicted ASMI. Because these thresholds were not developed specifically for anthropometrically predicted ASMI, this classification was treated as an epidemiological exposure definition rather than a clinical diagnosis of sarcopenia. Moreover, because the prediction equation incorporates body weight and circumferential measurements, the resulting classification may reflect overall body size in addition to skeletal muscle mass.

To assess dependence on the exposure specification, sensitivity analyses replaced binary LMM with continuous predicted ASMI derived from the Kawakami equation and from the alternative anthropometric equation developed by Wen et al. in Chinese adults [30]. Additional analyses adjusted separately for continuous body mass index (BMI) and underweight status, defined as BMI < 18.5 kg/m^2^.

#### 2.2.2. Overall Quality of Life and Self-Rated Health

Overall quality of life was assessed using a single global self-report item from the CLHLS. Responses were recorded on a five-category scale and coded so that higher values indicated better perceived quality of life. This item was specified as the primary outcome.

Self-rated health was assessed using a separate global self-report item with five ordered response categories and was similarly coded so that higher scores represented better perceived health. To evaluate the sensitivity of the findings to outcome specification, two additional outcomes were examined: self-rated health alone and a two-item composite calculated as the mean of the overall-QOL and self-rated-health items. The correlation between the two global items was also examined to characterize their empirical overlap.

#### 2.2.3. Sleep Quality

Sleep quality was assessed using a single self-reported CLHLS item: “How about the quality of your sleep?” Responses were recorded on a five-point scale and reverse-coded so that scores ranged from 1 (very poor) to 5 (very good), with higher scores indicating better perceived sleep quality [16,31]. The reverse-coded sleep score was treated as approximately continuous in the primary regression-based analysis. Because the response categories are ordinal, additional ordinal logistic models were used to evaluate the robustness of the associations without assuming equal spacing between adjacent response categories.

Both sleep quality and overall QOL were measured using single global subjective items administered during the same interview. These measures capture broad self-reported perceptions rather than multidimensional assessments of sleep or QOL. Their shared mode and timing of measurement may contribute to correlated responses and cannot distinguish domain-specific associations from common psychological or reporting influences.

#### 2.2.4. Core Covariates

Core covariates were selected a priori on the basis of substantive knowledge of factors potentially associated with the anthropometrically derived exposure, self-reported sleep quality, and overall QOL. The adjustment set was intended to account for measured demographic, socioeconomic, behavioral, and clinical differences, without implying that all confounding had been eliminated. The primary adjustment set included age; sex; residence (urban/rural); marital status (married/not currently married); education level (0, 1–6, or ≥7 years); self-reported economic status (good, average, or poor); co-residence (with household members, alone, or in an institution); current smoking; current drinking; regular exercise; hypertension; and diabetes.

Age was mean-centered and entered using both linear and quadratic terms to allow for nonlinear confounding across the wide age range. Multicategorical covariates were represented by k−1 indicator variables. Participants with no formal education, good economic status, and co-residence with household members served as the reference categories for education, economic status, and living arrangement, respectively.

**Figure 2:**
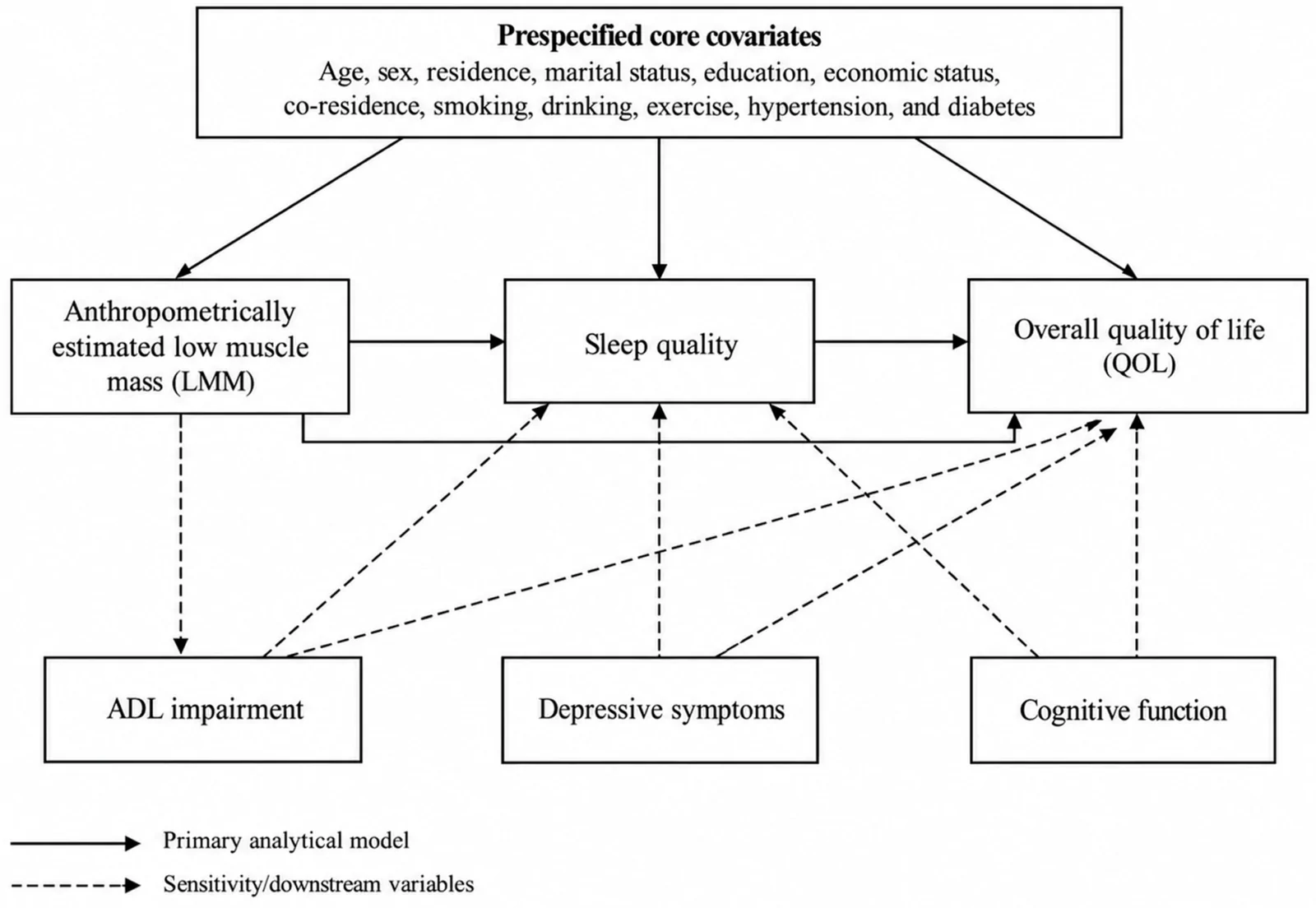
Analytical framework for the primary model and sensitivity analyses. Anthropometrically estimated low muscle mass (LMM), self-reported sleep quality, and overall quality of life (QOL) were specified as X, M, and Y, respectively. Solid arrows represent associations examined in the primary model with adjustment for prespecified core covariates, whereas dashed arrows indicate relationships considered in sensitivity analyses. Arrow directions reflect the analytical specification rather than established temporal or causal relationships. ADL, activities of daily living.

The primary analytical model, prespecified covariates, and sensitivity analyses are summarized in Figure 2.

#### 2.2.5. Additional Variables Used in Sensitivity Analyses

Activities of daily living (ADL) impairment was defined as difficulty with or need for assistance in at least one of six basic activities: bathing, dressing, toileting, transferring, continence, or eating. Because impaired physical functioning may occur downstream of reduced muscle health, ADL impairment was not included in the primary adjustment set and was instead evaluated in a same-sample sensitivity analysis [32,33].

Depressive symptoms were assessed using the 10-item Center for Epidemiologic Studies Depression Scale (CES-D-10) [34]. For the main depression sensitivity analysis, a modified nine-item score was calculated after excluding the sleep-related item to reduce direct content overlap with the sleep-quality intermediate variable. The conventional 10-item CES-D score was examined separately as a more stringent sensitivity analysis.

Cognitive function was assessed using the 30-point Chinese version of the Mini-Mental State Examination (MMSE) adopted in the CLHLS [35], with higher scores indicating better cognitive performance.

Respondent assistance was assessed using the interviewer-recorded item indicating whether another person helped the participant answer the questionnaire. One sensitivity analysis excluded respondents who received such assistance. A stricter analysis additionally required participants to have completed the relevant interview sections independently.

### 2.3. Statistical Analysis

Continuous variables are presented as means with standard deviations (SDs) and categorical variables as frequencies and percentages. Characteristics were compared according to LMM status using independent-samples t tests for continuous variables and Pearson chi-square tests for categorical variables. Pearson correlation coefficients were calculated for the bivariate associations among LMM, sleep quality, and overall QOL.

The primary analysis used PROCESS Model 4 to examine the regression-based statistical indirect association between anthropometrically estimated low muscle mass (LMM) and overall quality of life (QOL), with self-reported sleep quality specified as the intermediate variable. LMM, sleep quality, and overall QOL were designated as X, M, and Y, respectively, for model specification. Both regression equations were adjusted for the prespecified core covariates. The indirect association was calculated as the product of the exposure–sleep and sleep–QOL regression coefficients (a × b), with 95% confidence intervals estimated using 5000 bootstrap resamples. Unstandardized coefficients are reported. Because all three variables were measured during the same survey wave, this analysis was regarded as an exploratory statistical decomposition of concurrent associations, without establishing temporal precedence or causal mediation. A reverse-order model, specifying overall QOL as the intermediate variable and sleep quality as the outcome, was additionally examined to assess the dependence of the indirect association on the chosen model ordering.

Secondary exploratory conditional process analyses were conducted using PROCESS Model 59 to examine whether the indirect association varied according to sex or marital status. Sex and marital status were evaluated in separate models as moderators of the LMM–sleep, sleep–QOL, and direct LMM–QOL paths. Conditional indirect associations and indices of moderated mediation were estimated using 5000 bootstrap resamples. The marital-status analysis was additionally repeated with LMM-by-age and LMM-by-sex interaction terms included to distinguish marital-status-related heterogeneity from competing age- or sex-related effect modification. Because six primary interaction terms were examined across the sex and marital-status models, Holm adjustment was additionally applied as a multiplicity check.

Several sensitivity analyses assessed the dependence of the findings on outcome definition, exposure specification, model form, adjustment strategy, and respondent characteristics. Alternative outcome analyses used self-rated health and the two-item overall-QOL/self-rated-health composite in a common analytic sample. Ordinal logistic regression was used to examine the component associations when sleep quality and overall QOL were treated as ordered categorical variables. Alternative muscle-mass analyses used continuous Kawakami ASMI and continuous Wen ASMI. Additional models adjusted separately for continuous BMI and underweight. To further assess the impact of BMI adjustment, we compared the component regression coefficients before and after including continuous BMI and examined multicollinearity using variance inflation factors (VIFs). Further analyses adjusted for depressive symptoms, cognitive function, and ADL impairment; excluded respondents who received assistance; applied a strict self-response restriction; reversed the ordering of sleep quality and overall QOL; and estimated partially standardized effects.

Potential bias associated with complete-case analysis was evaluated in several complementary analyses. Variable-specific missingness and selected characteristics of participants included in and excluded from the final complete-case sample were first summarized. Multiple imputation using 50 imputed datasets was then performed among 14,088 participants with valid LMM information under a missing-at-random assumption conditional on variables included in the imputation framework. In addition, inverse-probability-of-complete-case weighting was applied to the complete-case sample, and weighted structural equation models (SEMs) with robust standard errors were compared with the unweighted complete-case model.

A separate sampling-weight sensitivity analysis incorporated the official 2018 CLHLS sampling weight (w_2018) together with robust standard errors. Because geographic cluster identifiers required to reconstruct the complete multistage survey design are unavailable in the public-use dataset, this analysis was interpreted as a sampling-weight sensitivity analysis rather than a fully specified complex-survey analysis.

As an exploratory assessment of sensitivity to unmeasured common causes of sleep quality and overall QOL, we applied a residual-correlation sensitivity framework based on the approach of Imai et al. [36]. The sensitivity parameter ρ represents a hypothetical correlation between the residuals of the specified intermediate-variable and outcome regressions. We examined how the estimated indirect association changed across assumed values of ρ and identified the values at which the confidence interval included zero and the point estimate reached zero. Because the data were cross-sectional and the assumptions required for causal mediation were not established, this analysis was interpreted as a model-assumption sensitivity exercise rather than validation of a causal indirect effect.

Primary regression and conditional-process analyses were conducted using IBM SPSS Statistics version 25.0 and PROCESS version 4.1. Ordinal, multiple-imputation, inverse-probability-weighted, structural-equation, and sampling-weighted analyses were conducted using Stata version 17. The residual-correlation sensitivity analysis was conducted in R using the mediation package. All statistical tests were two-sided, and *p* < 0.05 was considered statistically significant. For bootstrap-based indirect associations and indices of moderated mediation, statistical evidence was assessed using 95% bootstrap confidence intervals that did not include zero. Regression estimates, odds ratios, correlation coefficients, confidence limits, and *p* values were reported to three decimal places; *p* values <0.001 were reported as *p* < 0.001.

## 3. Results

### 3.1. Characteristics of Study Participants

Table 1 summarizes the characteristics of the 9366 participants included in the primary analysis. The mean age was 83.72 ± 11.47 years, 54.2% were women, and 57.9% lived in urban areas. Overall, 5753 participants (61.4%) were classified as having low muscle mass (LMM), whereas 3613 (38.6%) were classified as not having LMM. Compared with participants without LMM, those with LMM were older (87.12 ± 11.09 vs. 78.30 ± 9.90 years) and had a lower mean body mass index (20.43 ± 2.85 vs. 25.76 ± 3.68 kg/m^2^; both *p* < 0.001). Participants with LMM were also more likely to be women, live in rural areas, be not currently married, have no formal education, and report poor economic status, and were less likely to report current drinking and regular exercise (all *p* < 0.001). Co-residence differed modestly according to LMM status (*p* = 0.030), whereas current smoking did not (*p* = 0.322). Among participants with available ADL data, ADL impairment was more common in the LMM group than in the non-LMM group (23.85% vs. 12.39%, *p* < 0.001). Participants with LMM also reported slightly lower overall QOL scores (3.88 ± 0.78 vs. 3.97 ± 0.80) and sleep-quality scores (3.49 ± 0.97 vs. 3.58 ± 0.99; both *p* < 0.001).

### 3.2. Correlation Analysis

Pearson correlation coefficients are presented in Table 2. LMM was weakly negatively correlated with sleep quality (r = −0.049, *p* < 0.001) and overall QOL (r = −0.057, *p* < 0.001), whereas sleep quality was positively correlated with overall QOL (r = 0.256, *p* < 0.001).

### 3.3. Primary Indirect Association Analysis

**Figure 3:**
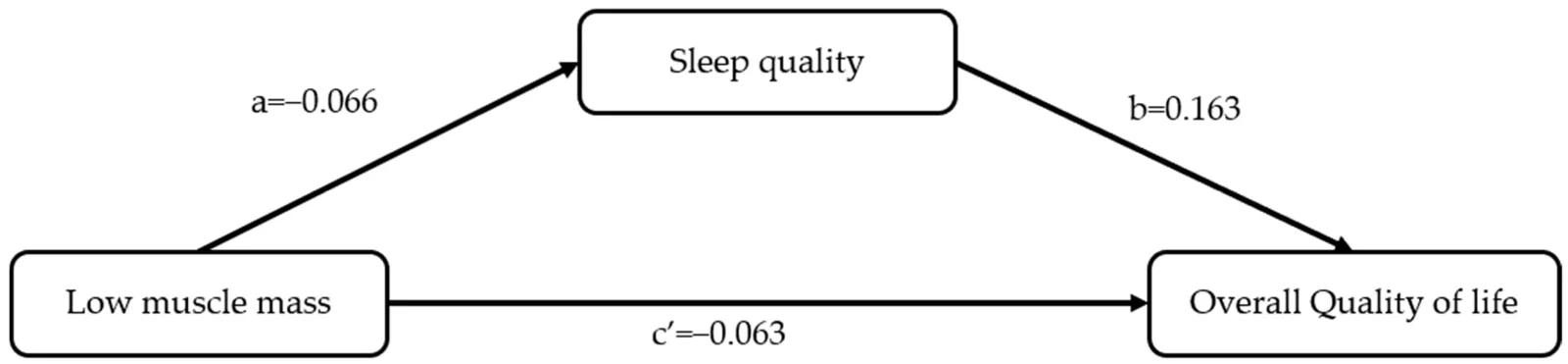
Primary indirect association model linking low muscle mass, sleep quality, and overall quality of life. Values are unstandardized regression coefficients from the adjusted primary model. Arrows indicate the analytical direction specified for the cross-sectional statistical decomposition.

The results of the primary analysis are presented in Table 3 and Figure 3. After adjustment for the prespecified core covariates, LMM was associated with poorer sleep quality (path a: B = −0.066, 95% CI, −0.110 to −0.022; *p* = 0.004). Sleep quality was positively associated with overall QOL after accounting for LMM and the covariates (path b: B = 0.163, 95% CI, 0.147 to 0.178; *p* < 0.001).

The total association between LMM and overall QOL was negative (c = −0.073, 95% CI, −0.108 to −0.039; *p* < 0.001). After sleep quality was included in the outcome model, the direct association remained negative (c′ = −0.063, 95% CI, −0.096 to −0.029; *p* < 0.001). The estimated indirect association through sleep quality was −0.011 (95% bootstrap CI, −0.018 to −0.003), with the bootstrap confidence interval excluding zero.

### 3.4. Secondary Exploratory Conditional Process Analyses

Detailed conditional process analyses are presented in Supplementary Table S1. The conditional indirect associations were similar in men and women, and the index of moderated mediation for sex included zero (index = −0.000, 95% bootstrap CI, −0.014 to 0.014), providing no evidence that the indirect association differed by sex.

In the base marital-status model, the index of moderated mediation was −0.015 (95% bootstrap CI, −0.030 to −0.002). After additional inclusion of LMM-by-age and LMM-by-sex interaction terms, the index was −0.013 (95% bootstrap CI, −0.030 to 0.003). Thus, the marital-status-related heterogeneity observed in the base model was not retained in the expanded specification. None of the six primary interaction tests remained below the 0.05 threshold after Holm adjustment (all adjusted *p* ≥ 0.086). These exploratory analyses did not provide consistent evidence of subgroup heterogeneity after accounting for the expanded model specification and multiple testing.

### 3.5. Sensitivity Analyses

Overall QOL and self-rated health were correlated (Spearman ρ = 0.484). Sensitivity analyses using alternative outcome definitions and model specifications produced associations in the same direction as the primary analysis (Supplementary Table S2). In the common analytic sample (*N* = 9357), the indirect association was −0.016 (95% bootstrap CI, −0.028 to −0.005) when self-rated health was used as the outcome and −0.014 (95% bootstrap CI, −0.023 to −0.004) when the two-item composite score was used. Treating sleep quality and overall QOL as ordinal outcomes also yielded consistent component associations: LMM was associated with lower odds of reporting better sleep quality (OR = 0.880, 95% CI, 0.808 to 0.958; *p* = 0.003) and lower odds of reporting better overall QOL after accounting for sleep quality (OR = 0.848, 95% CI, 0.774 to 0.929; *p* < 0.001).

Additional adjustment for depressive symptoms attenuated the estimated indirect association (Supplementary Table S3). In the CES-D-complete sample, the estimate changed from −0.011 (95% bootstrap CI, −0.019 to −0.003) to −0.006 (95% bootstrap CI, −0.011 to −0.002) after adjustment for the modified CES-D-9, which excluded the sleep-related item. Adjustment for the conventional CES-D-10 produced further attenuation. Adjustment for cognitive function produced little change (indirect association = −0.011, 95% bootstrap CI, −0.019 to −0.004), as did exclusion of respondents who received assistance (−0.010, 95% bootstrap CI, −0.018 to −0.002). Under the strict self-response restriction, the estimate remained similar in direction but was less precise (−0.008, 95% bootstrap CI, −0.016 to 0.000; Supplementary Table S3).

Results were also examined using alternative muscle-mass specifications and body-size adjustments (Supplementary Table S4). Continuous ASMI estimates derived from the Kawakami and Wen equations yielded indirect associations in the expected direction (0.004, 95% bootstrap CI, 0.001 to 0.007; and 0.012, 95% bootstrap CI, 0.008 to 0.017, respectively). Additional adjustment for underweight produced an indirect estimate of −0.009 (95% bootstrap CI, −0.017 to −0.001). In contrast, adjustment for continuous BMI markedly attenuated the estimated indirect association from −0.011 (95% bootstrap CI, −0.018 to −0.003) to −0.001 (95% bootstrap CI, −0.010 to 0.008). The LMM–sleep coefficient decreased from −0.066 to −0.006, and the conditional LMM–QOL coefficient decreased in magnitude from −0.063 to −0.017. The variance inflation factor for LMM was 1.851 in the BMI-adjusted models, providing no evidence of severe linear multicollinearity affecting the LMM coefficient (Supplementary Table S10).

In the ADL-complete sample (*N* = 9022), additional adjustment for ADL impairment produced essentially unchanged estimates (indirect association = −0.010, 95% bootstrap CI, −0.018 to −0.003; Supplementary Table S5). A reverse-order model specifying overall QOL as the intermediate variable also yielded a non-zero indirect association (−0.020, 95% bootstrap CI, −0.030 to −0.011). In the partially standardized primary model, the indirect association corresponded to −0.014 SD (95% bootstrap CI, −0.023 to −0.004).

Of the 15,771 age-eligible participants, 9366 (59.4%) were included in the primary complete-case analysis, while 6405 (40.6%) were excluded. Excluded participants were older and were more likely to be women and rural residents than those included in the primary sample. Variable-specific missingness was greatest for education, diabetes, anthropometric information required for LMM estimation, overall QOL, sleep quality, and hypertension (Supplementary Table S6).

The primary estimate was similar under alternative approaches to missing data and complete-case selection (Supplementary Table S7). Multiple imputation using 50 imputed datasets among 14,088 participants with valid LMM information yielded an indirect association of −0.012 (95% CI, −0.018 to −0.006), and the inverse-probability-weighted SEM yielded an estimate of −0.010 (95% CI, −0.018 to −0.003). Finally, analysis incorporating the official 2018 CLHLS sampling weight among 9266 participants produced an indirect association of −0.015 (95% CI, −0.025 to −0.004; *p* = 0.006), with the direction of the component and total associations remaining consistent with the unweighted analysis (Supplementary Table S8). These analyses cannot exclude bias arising from unmeasured determinants of sample inclusion.

Sensitivity to hypothetical residual dependence between the sleep-quality and QOL regression equations was evaluated using a residual-correlation analysis (Supplementary Table S9). The point estimate of the indirect association was reduced to zero at ρ = 0.210, while the 95% confidence interval first included zero at approximately ρ = 0.190. The corresponding residual-variance R^2^ and total-variance R^2^ products were 0.044 and 0.035, respectively. These findings indicate sensitivity of the statistical indirect association to assumptions concerning unmeasured common causes.

## 4. Discussion

In this cross-sectional analysis of 9366 Chinese adults aged 65 years or older, anthropometrically estimated low muscle mass (LMM) was associated with slightly poorer self-reported sleep quality and overall quality of life (QOL). Importantly, the estimated statistical indirect association involving sleep quality was small in the primary analysis, and was substantially attenuated after adjustment for continuous body mass index, highlighting its sensitivity to overall body size. Additional adjustment for depressive symptoms further attenuated the estimate, while a reverse-order model also yielded a non-zero indirect association. Sensitivity analysis indicated that the indirect estimate depended on assumptions about unmeasured common causes of sleep quality and QOL. Taken together, these findings characterize a set of concurrent, model-dependent associations rather than identifying a muscle-specific mechanism or a causal pathway operating through sleep quality.

The observed associations are broadly consistent with previous research linking reduced muscle health to poorer QOL and sleep-related outcomes. Systematic reviews and population-based studies have reported poorer health-related QOL among individuals with sarcopenia or reduced muscle health [4,5,6]. Studies examining sleep characteristics have also identified associations with muscle mass, muscle strength, and probable sarcopenia in older adults [10,11,12]. Furthermore, sleep quality has been consistently associated with QOL in ageing populations [13,14]. These findings provide a rationale for examining muscle-related characteristics, sleep quality, and perceived well-being together. Nevertheless, the populations, measurement approaches, and analytical designs used in previous studies differ from those of the present analysis. Consequently, consistency in the direction of associations should not be interpreted as confirmation of a shared biological mechanism.

The Wilson–Cleary conceptual model and its subsequent refinement by Ferrans et al. provide a broader framework for considering relationships among physiological characteristics, symptom experiences, functional status, and subjective evaluations of health and QOL [8,9]. Within this framework, anthropometrically estimated muscle mass, perceived sleep quality, and overall QOL represent distinct but potentially related health domains. However, the framework does not establish the temporal ordering of these particular measures in the CLHLS. Their concurrent associations may arise from several overlapping biological, functional, psychological, and social processes. Accordingly, the statistical indirect association estimated in this study should be viewed as one descriptive representation of these relationships rather than as evidence of a specific sequence of effects.

The magnitude of the estimated associations is important for their interpretation. The partially standardized indirect association corresponded to approximately −0.014 standard deviations in overall QOL, indicating a very small statistical component relative to the variability of the outcome. Although the large sample allowed relatively precise estimation, a confidence interval excluding zero does not establish clinical importance. Moreover, the single-item QOL measure has no established minimally important difference applicable to this analysis, limiting the translation of the coefficient into a clinically meaningful change. The results should therefore be interpreted in terms of their statistical magnitude and uncertainty rather than as evidence of a clinically consequential sleep-related mechanism.

Sensitivity analyses using alternative outcome definitions and model specifications provided additional information about the measurement dependence of the findings. Replacing overall QOL with self-rated health or a two-item QOL/health composite yielded statistical indirect associations in the same direction. Ordinal regression analyses also produced component associations consistent with the primary models, suggesting that the observed direction was not solely attributable to treating five-category subjective responses as continuous variables. Similarly, continuous ASMI estimates based on the Kawakami and Wen anthropometric equations were associated with sleep quality and QOL in the expected direction [27,30]. However, these alternative specifications do not eliminate measurement error, establish clinical validity of the predicted muscle-mass classification, or resolve the shared subjective measurement characteristics of sleep quality and QOL.

The pronounced attenuation after continuous BMI adjustment is particularly important for interpreting the anthropometrically derived LMM exposure. The Kawakami prediction equation incorporates body weight, height, waist circumference, and calf circumference, while BMI also reflects weight relative to height [27]. Thus, predicted ASMI and BMI share body-size information, and adjustment for BMI changes the association being estimated. Although the application of established ASMI thresholds facilitates comparison with previous studies using anthropometric estimates [3,28,29], these thresholds were not specifically validated for the predicted ASMI values in the present sample. Adjustment for underweight produced relatively modest attenuation, whereas adjustment for continuous BMI reduced the estimated indirect association to near zero. The variance inflation factor for LMM was 1.851 in the BMI-adjusted component regressions, suggesting that severe linear multicollinearity was not evident. However, this diagnostic does not establish the construct validity of anthropometrically estimated muscle mass or distinguish confounding by overall body size from adjustment for shared measurement components. Because the indirect association was reduced to near zero after continuous BMI adjustment, the findings cannot be considered robust evidence of an association attributable specifically to skeletal muscle mass. Future studies using directly measured body composition are needed to determine whether similar associations persist independently of overall body size.

Psychological factors may also contribute to the observed relationships. Adjustment for depressive symptoms attenuated the statistical indirect association, including when the modified CES-D-9 score was used to exclude the sleep-related item. Greater attenuation following adjustment for the conventional CES-D-10 requires additional caution because that score includes sleep-related content. Previous CLHLS research has reported an association between anthropometrically estimated LMM and depressive symptoms [19], while another study identified interrelationships among depressive symptoms, cognitive performance, and QOL in older adults [37]. The attenuation following depressive-symptom adjustment suggests that the estimated indirect association is sensitive to psychological covariate specification. However, depressive symptoms may represent a shared correlate, a potential common cause, or a consequence of the health domains examined, and their temporal role cannot be determined from these data. In addition, the conventional CES-D-10 includes a sleep-related item, introducing conceptual overlap with the sleep-quality measure. Consequently, neither the attenuation after psychological adjustment nor the remaining non-zero estimate after CES-D-9 adjustment can establish or exclude a distinct sleep-specific mechanism.

Sleep quality and overall QOL were assessed using single-item self-report measures collected during the same interview. Although these items provide global assessments of perceived sleep quality and QOL, they do not capture the multidimensional nature of either construct. Their concurrent assessment using a similar reporting method also raises the possibility of shared reporting tendencies, psychological symptom burden, and other common influences on subjective health evaluations. The attenuation of the estimated indirect association after adjustment for depressive symptoms is consistent with this concern, although it does not establish the extent to which shared psychological factors account for the observed association. Furthermore, alternative outcome definitions, ordinal regression analyses, and restrictions based on respondent assistance cannot isolate or eliminate common-method variance. Consequently, the observed sleep–QOL association cannot be confidently attributed to a distinct sleep-specific mechanism.

The treatment of ADL impairment was also examined because functional limitation may occur downstream of reduced muscle health. Longitudinal evidence indicates that muscle mass, strength, and physical performance can predict subsequent ADL limitations [32], and adjustment for downstream variables may introduce overadjustment bias [33]. ADL impairment was therefore excluded from the prespecified primary adjustment set and examined separately. Additional adjustment within the same ADL-complete sample produced little change in the estimated indirect association. Nevertheless, the temporal position of ADL impairment relative to the anthropometric exposure, sleep quality, and QOL cannot be established from the cross-sectional data. This analysis should therefore be interpreted as an alternative covariate specification rather than as a test that determines the causal role of functional impairment.

The reverse-order analysis further illustrates the limitations of temporal interpretation. When overall QOL was specified as the intermediate variable and sleep quality as the outcome, a non-zero statistical indirect association was also observed. This alternative result does not establish a causal pathway from QOL through sleep, just as the primary specification does not establish mediation through sleep quality. Longitudinal evidence suggests that sleep characteristics may precede changes in muscle-related outcomes [15] and that sleep quality and life satisfaction may exhibit reciprocal associations [16]. Methodological studies have also demonstrated that cross-sectional mediation analyses may provide misleading representations of longitudinal processes when temporal ordering is unknown [17,18]. The present regression-based decomposition is therefore conditional on the selected analytical ordering and cannot determine the direction of the underlying relationships.

The residual-correlation sensitivity analysis provides an additional qualification. Under the framework described by Imai et al. [36], the confidence interval for the estimated indirect association first included zero at approximately ρ = 0.190, and the point estimate reached zero at ρ = 0.210. These values represent hypothetical residual correlations between the specified regression equations rather than measured correlations attributable to a particular unobserved factor. The findings indicate that the statistical indirect association is sensitive to assumptions concerning unmeasured common causes of sleep quality and QOL. Because the assumptions necessary for causal mediation are not established in this cross-sectional setting, this analysis should not be interpreted as validating or quantifying a causal indirect effect.

The exclusion of 40.6% of age-eligible participants represents an important potential source of selection bias. Excluded participants differed from those included in the primary analysis in age, sex, and residence, raising concerns about the representativeness of the complete-case sample. Although multiple imputation, inverse-probability-of-complete-case weighting, and sampling-weight sensitivity analyses yielded indirect estimates similar in direction and magnitude to the primary estimate, these findings do not establish the absence of selection bias. In particular, multiple imputation was restricted to participants with valid information for constructing the anthropometric exposure, and both imputation and complete-case weighting relied on assumptions about missingness or selection that cannot be fully verified. Furthermore, the sampling-weight analysis could not account for the complete multistage survey design because the required geographic cluster identifiers were unavailable in the public-use data [26]. The magnitude and direction of any remaining selection bias therefore remain uncertain, and the findings should not be assumed to generalize to all age-eligible CLHLS participants or the broader Chinese older-adult population.

The exploratory conditional process analyses provided limited evidence of consistent heterogeneity by sex or marital status. The estimated indirect association was similar in men and women, and the corresponding index of moderated mediation included zero. Although the initial marital-status analysis suggested heterogeneity, this finding was not maintained after additional interaction terms were incorporated. These results should be considered in the context of previous research documenting sex-related differences in ageing-associated body composition and sleep characteristics [22,23], as well as associations of marital relationships and social support with sleep in older adults [24,25]. Moreover, sex is incorporated into the anthropometric ASM equation and sex-specific thresholds were used to define LMM, which further complicates interpretation of apparent sex-related differences. The subgroup analyses should therefore be regarded as exploratory rather than evidence of distinct biological or psychosocial pathways.

Several strengths of this study merit recognition. The analysis included a large sample of older Chinese adults and examined muscle-related characteristics, sleep quality, and overall QOL within a common statistical framework. It also incorporated alternative outcome definitions, continuous anthropometric muscle-mass estimates, ordinal regression, psychological and cognitive adjustments, respondent restrictions, alternative missing-data approaches, sampling weights, and model-assumption sensitivity analyses. These analyses help characterize the extent to which the observed findings depend on specific analytical choices. Nevertheless, they cannot overcome the principal limitations of the available data: the absence of directly measured muscle mass, reliance on single-item subjective assessments, simultaneous measurement of the principal variables, substantial complete-case exclusion, and potential residual confounding. The cross-sectional design also prevents identification of temporal or causal mediation.

The principal contribution of this study lies in characterizing the associations among anthropometrically estimated muscle mass, perceived sleep quality, and overall QOL, while systematically examining their dependence on measurement definitions, body-size adjustment, psychological covariates, model ordering, and missing-data assumptions. The findings underscore the importance of distinguishing statistically detectable associations from clinically meaningful or causally identifiable mechanisms. They do not establish that improving sleep quality would improve QOL among individuals classified as having LMM, nor do they provide sufficient evidence to recommend a sleep-focused intervention on the basis of the estimated indirect association. Future longitudinal investigations should incorporate directly measured muscle mass, validated multidimensional sleep and QOL assessments, and repeated measurements of relevant psychological and functional characteristics. Such studies would be better positioned to evaluate temporal relationships, distinguish muscle-specific associations from broader body-size effects, and establish the clinical relevance of the observed patterns.

## 5. Conclusions

In this cross-sectional analysis of older Chinese adults, anthropometrically estimated low muscle mass (LMM) was associated with slightly poorer self-reported sleep quality and overall quality of life (QOL). However, the estimated indirect association involving sleep quality was small and was substantially attenuated after adjustment for continuous body mass index (BMI), highlighting its dependence on overall body size. Further attenuation following depressive-symptom adjustment, the non-zero indirect association in the reverse-order model, and sensitivity to unmeasured confounding further limited support for a distinct sleep-related pathway. The use of single-item subjective measures and substantial complete-case exclusions also constrain interpretation and generalizability. Overall, these findings characterize concurrent, model-dependent associations rather than a muscle-specific or causally mediated mechanism. Longitudinal studies incorporating directly measured body composition, validated multidimensional sleep and QOL assessments, and repeated measurements are needed to clarify the temporal relationships and clinical relevance of these findings.

## Figures and Tables

**Table 1:** Characteristics of study participants according to low muscle mass status in the final primary analytic sample (*N* = 9366).

Variables	Total	LMM		*p* Value
	(*N* = 9366)	No (*n* = 3613)	Yes (*n* = 5753)	
Age, years, M ± SD	83.72 ± 11.47	78.30 ± 9.90	87.12 ± 11.09	<0.001
BMI, kg/m^2^	22.49 ± 4.12	25.76 ± 3.68	20.43 ± 2.85	<0.001
Sex, *n* (%)				<0.001
Male	4288 (45.78)	2013 (55.72)	2275 (39.54)	
Female	5078 (54.22)	1600 (44.28)	3478 (60.46)	
Residence, *n* (%)				<0.001
Urban	5420 (57.87)	2269 (62.80)	3151 (54.77)	
Rural	3946 (42.13)	1344 (37.20)	2602 (45.23)	
Marital status, *n* (%)				<0.001
Not currently married	5065 (54.08)	1397 (38.67)	3668 (63.76)	
Married	4301 (45.92)	2216 (61.33)	2085 (36.24)	
Education level, years, *n* (%)				<0.001
0	4195 (44.79)	1037 (28.70)	3158 (54.89)	
1–6	3211 (34.28)	1403 (38.83)	1808 (31.43)	
≥7	1960 (20.93)	1173 (32.47)	787 (13.68)	
Economic status, *n* (%)				<0.001
Good	1891 (20.19)	810 (22.42)	1081 (18.79)	
Average	6573 (70.18)	2540 (70.30)	4033 (70.10)	
Poor	902 (9.63)	263 (7.28)	639 (11.11)	
Co-residence, *n* (%)				0.030
With household members	7575 (80.88)	2968 (82.15)	4607 (80.08)	
Living alone	1524 (16.27)	542 (15.00)	982 (17.07)	
Living in an institution	267 (2.85)	103 (2.85)	164 (2.85)	
Current smoking, *n* (%)				0.322
Yes	1484 (15.84)	590 (16.33)	894 (15.54)	
No	7882 (84.16)	3023 (83.67)	4859 (84.46)	
Current drinking, *n* (%)				<0.001
Yes	1448 (15.46)	681 (18.85)	767 (13.33)	
No	7918 (84.54)	2932 (81.15)	4986 (86.67)	
Regular exercise, *n* (%)				<0.001
Yes	3208 (34.25)	1564 (43.29)	1644 (28.58)	
No	6158 (65.75)	2049 (56.71)	4109 (71.42)	
Hypertension, *n* (%)				<0.001
Yes	4050 (43.24)	1942 (53.75)	2108 (36.64)	
No	5316 (56.76)	1671 (46.25)	3645 (63.36)	
Diabetes, *n* (%)				<0.001
Yes	1058 (11.30)	618 (17.10)	440 (7.65)	
No	8308 (88.70)	2995 (82.90)	5313 (92.35)	
ADL impairment, *n*/*N* (%)				<0.001
Yes	1754/9022 (19.44)	430/3470 (12.39)	1324/5552 (23.85)	
No	7268/9022 (80.56)	3040/3470 (87.61)	4228/5552 (76.15)	
Overall QOL score, M ± SD	3.92 ± 0.79	3.97 ± 0.80	3.88 ± 0.78	<0.001
Sleep quality score, M ± SD	3.52 ± 0.98	3.58 ± 0.99	3.49 ± 0.97	<0.001

Data are presented as mean  ±  standard deviation (SD) or frequency (percentage); Activities of daily living (ADL) impairment was available for 9022 participants (3470 without LMM and 5552 with LMM); percentages for ADL impairment therefore use available-case denominators. Body mass index (BMI) is presented descriptively and was not included in the primary adjustment set. LMM, low muscle mass; BMI, body mass index; ADL, activities of daily living; QOL, quality of life.

**Table 2:** Correlations among low muscle mass, sleep quality, and quality of life.

Variable	Low Muscle Mass	Sleep Quality	Overall QOL
Low muscle mass	1		
Sleep quality	−0.049 ***	1	
Overall QOL	−0.057 ***	0.256 ***	1

Pearson correlation coefficients are presented; *** *p* < 0.001. QOL, quality of life.

**Table 3:** Direct and indirect associations between low muscle mass and overall quality of life through sleep quality.

Effect	B (95% CI)	SE	*p* Value
LMM → sleep quality (a)	−0.066 (−0.110 to −0.022)	0.023	0.004
Sleep quality →Overall QOL (b)	0.163 (0.147 to 0.178)	0.008	<0.001
Total association (c)	−0.073 (−0.108 to −0.039)	0.018	<0.001
Direct association (c′)	−0.063 (−0.096 to −0.029)	0.017	<0.001
Indirect association (a × b)	−0.011 (−0.018 to −0.003)	0.004	—

B, unstandardized regression coefficient; CI, confidence interval; SE, standard error; LMM, low muscle mass; QOL, quality of life. For the indirect association, SE and 95% CI were estimated using 5000 bootstrap resamples. Models were adjusted for the core covariates.

## Data Availability

The datasets used and/or analyzed during the current study are available through the Chinese Longitudinal Healthy Longevity Survey (CLHLS) repository on the Peking University Open Research Data Platform (https://opendata.pku.edu.cn/dataverse/CHADS) (accessed on15 September 2026), subject to the repository’s registration and access procedures.

## Supplementary Materials

The following supporting information can be downloaded at: https://www.mdpi.com/article/10.3390/healthcare14213762/s1, Table S1: Exploratory conditional process analyses by sex and marital status; Table S2: Sensitivity analyses using alternative outcome definitions and ordinal model specifications; Table S3: Sensitivity analyses for depressive symptoms, cognitive function, and respondent assistance; Table S4: Sensitivity analyses using alternative muscle-mass specifications and adjustment for body size; Table S5: Sensitivity analyses for ADL adjustment, reverse-order model specification, and standardized effect size; Table S6: Participant selection characteristics and missing-data profile for the primary complete-case analysis; Table S7: Sensitivity analyses using alternative approaches to missing data and complete-case selection; Table S8: Sampling-weight sensitivity analysis using the official 2018 CLHLS weight; Table S9: Sensitivity of the indirect association to unmeasured mediator–outcome confounding; Table S10: Component regression coefficients and collinearity diagnostics before and after adjustment for continuous BMI.

## Abbreviations

The following abbreviations are used in this manuscript:

ADL	Activities of daily living
ASM	Appendicular skeletal muscle mass
ASMI	Appendicular skeletal muscle mass index
BMI	Body mass index
CES-D	Center for Epidemiologic Studies Depression Scale
MMSE	Mini-Mental State Examination
CLHLS	Chinese Longitudinal Healthy Longevity Survey
CI	Confidence interval
LMM	Low muscle mass
DXA	Dual-energy X-ray absorptiometry
QOL	Quality of Life
SD	Standard deviation
OR	Odds ratio
SEM	Structural equation model
VIF	Variance Inflation factor
