# Supplementary material for: Low Muscle Mass, Sleep Quality, and Quality of Life Among Older Chinese Adults: A Cross-Sectional Study
Source: Healthcare (Basel). 2026 Nov 4;14(21):3762. doi: 10.3390/healthcare14213762 (PMC13762947; doi:10.3390/healthcare14213762)
Supplement: Supplementary file 1 [file healthcare-14-03762-s001.zip › healthcare-4599396-supplementary.pdf]

Supplementary material

All effect estimates and confidence limits are rounded to three decimal places; statistical inference was based on unrounded estimates.

Table S1. Exploratory conditional process analyses by sex and marital status.

Panel A. Conditional indirect associations and indices of moderated mediation

Moderator / model	Subgroup	N	Conditional indirect association	95% bootstrap CI	Index of moderated mediation	95% bootstrap CI
Sex	Male	9,366	−0.011	−0.021 to −0.001	−0.000	−0.014 to 0.014
	Female		−0.011	−0.021 to −0.001		
Marital status, base model	Not currently married	9,366	−0.003	−0.013 to 0.007	−0.015	−0.030 to −0.002
	Married		−0.019	−0.029 to −0.008		
Marital status, expanded model	Not currently married	9,366	−0.002	−0.015 to 0.011	−0.013	−0.030 to 0.003
	Married		−0.015	−0.028 to −0.003		

Panel B. Interaction tests in the conditional process models.

Model	Interaction term	P value
Sex	LMM × sex → sleep quality	0.886
Sex	LMM × sex → overall QOL	0.727
Sex	Sleep quality × sex → overall QOL	0.600
Marital status, base model	LMM × marital status → sleep quality	0.042
Marital status, base model	LMM × marital status → overall QOL	0.014
Marital status, base model	Sleep quality × marital status → overall QOL	0.196
Marital status, expanded model	LMM × marital status → sleep quality	0.130
Marital status, expanded model	LMM × marital status → overall QOL	0.099
Marital status, expanded model	Sleep quality × marital status → overall QOL	0.199
Marital status, expanded model	LMM × age → sleep quality	0.497
Marital status, expanded model	LMM × sex → sleep quality	0.597
Marital status, expanded	LMM × age → overall QOL	0.333

model		
Marital status, expanded model	LMM × sex → overall QOL	0.918

Note: Conditional process analyses were estimated using PROCESS Model 59 with 5,000 bootstrap resamples. Sex was coded as 0 = male and 1 = female; marital status was coded as 0 = not currently married and 1 = married. The expanded marital-status model additionally included LMM-by-age and LMM-by-sex interaction terms. Conditional indirect associations are reported with bootstrap confidence intervals. Evidence of heterogeneity in the indirect association was assessed using the bootstrap confidence interval for the index of moderated mediation. All analyses were exploratory and were adjusted for the prespecified core covariates, except that the moderator itself was not additionally entered as an ordinary covariate. Raw interaction P values are presented; none of the six primary interaction tests remained below 0.05 after Holm adjustment. LMM, low muscle mass; QOL, quality of life; CI, confidence interval.

Table S2. Sensitivity analyses using alternative outcome definitions and ordinal model specifications.

Panel A. Alternative outcome definitions in a common analytic sample.

Outcome	N	a: LMM → Sleep	b: Sleep → Outcome	Direct association (c′)	Indirect association	95% bootstrap CI
Overall QOL	9,357	−0.065	0.163	−0.063	−0.011	−0.018 to −0.003
Self-rated health	9,357	−0.065	0.252	−0.090	−0.016	−0.028 to −0.005
Two-item composite score	9,357	−0.065	0.207	−0.077	−0.014	−0.023 to −0.004

Note: Panel A compares alternative outcome definitions in the same analytic sample (N = 9,357), thereby holding sample composition constant across models. Higher scores represent better overall QOL, better self-rated health, and better sleep quality. LMM, low muscle mass; QOL, quality of life; CI, confidence interval.

Panel B. Ordinal logistic sensitivity analyses.

Ordinal outcome	Predictor / contrast	OR	95% CI	P value
Sleep quality	LMM	0.880	0.808 to 0.958	0.003
Overall QOL	LMM	0.848	0.774 to 0.929	<0.001
Overall QOL	Sleep quality: 2 vs. 1	1.167	0.840 to 1.620	0.357
Overall QOL	Sleep quality: 3 vs. 1	1.407	1.027 to 1.927	0.033
Overall QOL	Sleep quality: 4 vs. 1	2.000	1.463 to 2.734	<0.001
Overall QOL	Sleep quality: 5 vs. 1	5.425	3.898 to 7.551	<0.001

Note: Joint test for sleep-quality categories in the overall-QOL model: $\chi^2(4) = 434.670$, $P < 0.001$. Panel B treats the five-level sleep-quality and overall-QOL variables as ordered categorical outcomes. The ordinal analyses were used to evaluate the robustness of the component associations and were not interpreted as estimating an ordinal indirect effect. All models were adjusted for the prespecified core covariates. LMM, low muscle mass; QOL, quality of life; OR, odds ratio; CI, confidence interval.

Table S3. Sensitivity analyses for depressive symptoms, cognitive function, and respondent assistance.

Analysis	N	Indirect association	95% bootstrap CI
Primary model	9,366	−0.011	−0.018 to −0.003
CES-D-complete baseline	8,329	−0.011	−0.019 to −0.003
Additional adjustment for modified CES-D-9	8,329	−0.006	−0.011 to −0.002
Additional adjustment for conventional CES-D-10	8,329	−0.002	−0.004 to −0.000
MMSE-complete baseline	8,829	−0.012	−0.020 to −0.005
Additional adjustment for MMSE-30	8,829	−0.011	−0.019 to −0.004
Excluding respondents who received assistance	6,651	−0.010	−0.018 to −0.002
Strict self-response restriction	6,028	−0.008	−0.016 to 0.000

Note: All analyses used the primary exposure, intermediate variable, outcome, and prespecified core adjustment set unless otherwise specified. CES-D-complete and MMSE-complete baseline models were re-estimated in the corresponding complete subsamples before adding the respective sensitivity covariate. The modified CES-D-9 excludes the sleep-related CES-D item, whereas the conventional CES-D-10 includes this item and therefore has conceptual overlap with the sleep-quality intermediate variable. The assisted-response sensitivity analysis excluded participants for whom assistance with questionnaire responses was reported. The strict self-response analysis additionally required the participant to be able to complete the relevant interview sections independently. Indirect associations and 95% CIs were estimated using 5,000 bootstrap resamples. CES-D, Center for Epidemiologic Studies Depression Scale; MMSE, Mini-Mental State Examination; CI, confidence interval.

Table S4. Sensitivity analyses using alternative muscle-mass specifications and adjustment for body size.

Analysis	N	a: Exposure → Sleep	b: Sleep → QOL	Direct association (c′)	Indirect association	95% bootstrap CI
Primary binary LMM	9,366	−0.066	0.163	−0.063	−0.011	−0.018 to −0.003
Continuous Kawakami ASMI	9,366	0.023	0.163	0.022	0.004	0.001 to 0.007
Continuous Wen ASMI	9,366	0.077	0.161	0.057	0.012	0.008 to 0.017
LMM + continuous BMI	9,366	−0.006	0.162	−0.017	−0.001	−0.010 to 0.008
LMM + underweight	9,366	−0.053	0.162	−0.051	−0.009	−0.017 to −0.001

Note: The primary exposure was binary LMM. Continuous ASMI analyses replaced binary LMM with continuously estimated ASMI derived from the Kawakami or Wen anthropometric equations; therefore, positive coefficients indicate associations of greater muscle mass with better sleep quality and QOL. The BMI and underweight models retained binary LMM and added continuous BMI or underweight status (BMI <18.5 kg/m^2), respectively, to the adjustment set. BMI and anthropometrically estimated muscle mass share weight and height components; the BMI-adjusted analysis should therefore be interpreted as a stringent shared-component sensitivity analysis. All models included the prespecified core covariates. LMM, low muscle mass; ASMI, appendicular skeletal muscle mass index; BMI, body mass index; QOL, quality of life; CI, confidence interval.

Table S5. Sensitivity analyses for ADL adjustment, reverse-order model specification, and standardized effect size.

Panel A. Additional adjustment for ADL impairment.

Model	N	a: LMM → Sleep	b: Sleep → QOL	Direct association (c′)	Indirect association	95% bootstrap CI
ADL-complete baseline	9,022	−0.061	0.164	−0.059	−0.010	−0.018 to −0.003
Additional adjustment for ADL impairment	9,022	−0.062	0.164	−0.059	−0.010	−0.018 to −0.003

Panel B. Reverse-order model.

Path / effect	B	P value	95% bootstrap CI
LMM → overall QOL	−0.073	<0.001	—
Overall QOL → sleep quality	0.269	<0.001	—
Direct LMM → sleep quality association	−0.046	0.038	—
Indirect association through overall QOL	−0.020	—	−0.030 to −0.011

Panel C. Partially standardized primary model.

Path / effect	Standardized coefficient	P value	95% bootstrap CI
LMM → standardized sleep quality	−0.067 SD	0.004	—
Standardized sleep quality → standardized QOL	0.202	<0.001	—
Direct LMM → standardized QOL association	−0.079 SD	<0.001	—
Standardized indirect association	−0.014 SD	—	−0.023 to −0.004

Note: Panel A uses the same 9,022 participants with complete ADL data in both models, allowing changes associated with ADL adjustment to be separated from changes in sample composition. Panel B reverses the ordering of sleep quality and overall QOL as a model-specification sensitivity analysis; it is not intended to establish reverse causality. Panel C reports a partially standardized analysis in which sleep quality and overall QOL were standardized while binary LMM remained unstandardized. Indirect associations were estimated using 5,000 bootstrap resamples. ADL, activities of daily living; LMM, low muscle mass; QOL, quality of life; SD, standard deviation; CI, confidence interval.

Table S6. Participant selection characteristics and missing-data profile for the primary complete-case analysis.

Panel A. Missing-data profile for variables used in the primary analysis.

Variable	Available n	Missing n	Missing %
Age	15,771	0	0.00
Sex	15,771	0	0.00
Residence	15,771	0	0.00
Marital status	15,517	254	1.61
Education level	13,437	2,334	14.80
Economic status	15,493	278	1.76
Co-residence	15,457	314	1.99
Current smoking	15,518	253	1.60
Current drinking	15,423	348	2.21
Regular exercise	15,433	338	2.14
Hypertension	14,495	1,276	8.09
Diabetes	13,943	1,828	11.59
Anthropometric data required for LMM estimation	14,088	1,683	10.67
Overall QOL	14,345	1,426	9.04
Sleep quality	14,392	1,379	8.74

Panel B. Included versus excluded participants.

Characteristic	Included (N = 9,366)	Excluded (N = 6,405)	P value
Age, years, mean ± SD	83.72 ± 11.47	88.34 ± 11.21	<0.001
Sex, n (%)			<0.001
Male	4,288 (45.78)	2,581 (40.30)	
Female	5,078 (54.22)	3,824 (59.70)	
Residence, n (%)			<0.001
Urban	5,420 (57.87)	3,317 (51.79)	
Rural	3,946 (42.13)	3,088 (48.21)	

Note: Panel A summarizes availability and missingness for the variables used to construct the primary exposure, intermediate variable, outcome, and prespecified core adjustment set among all 15,771 eligible participants. Panel B compares participants included in the final primary complete-case analysis with eligible participants aged ≥65 years who were excluded from that analysis. Missingness across variables was overlapping; therefore, variable-specific missing counts should not be summed. Derived variables and variables used only in secondary sensitivity analyses are not listed separately. LMM, low muscle mass; QOL, quality of life.

Table S7. Sensitivity analyses using alternative approaches to missing data and complete-case selection.

Analysis	N	a: LMM → Sleep	b: Sleep → QOL	Direct association (c′)	Indirect association	95% CI for indirect association	Total association (c)
Complete-case unweighted SEM	9,366	−0.066	0.163	−0.063	−0.011	−0.018 to −0.003	−0.073
Multiple imputation, 50 datasets	14,088	−0.073	0.163	−0.072	−0.012	−0.018 to −0.006	−0.084
Inverse-probability-weighted SEM	9,366	−0.064	0.162	−0.060	−0.010	−0.018 to −0.003	−0.070

Note: The complete-case unweighted SEM served as the reference analysis. Multiple imputation used 50 imputed datasets among 14,088 participants with valid LMM information, with missing outcome, sleep-quality, and core-covariate data imputed under a missing-at-random framework. The inverse-probability-weighted analysis reweighted complete cases according to observed predictors of complete-case inclusion and used robust standard errors. These analyses evaluate robustness to alternative approaches for handling missing observations and observed complete-case selection but do not eliminate the possibility of bias related to unmeasured determinants of missingness. SEM, structural equation modeling; MI, multiple imputation; LMM, low muscle mass; QOL, quality of life; CI, confidence interval.

Table S8. Sampling-weight sensitivity analysis using the official 2018 CLHLS weight.

Effect	B	95% CI	P value
LMM → sleep quality (a)	−0.088	−0.150 to −0.026	0.005
Sleep quality → overall QOL (b)	0.168	0.142 to 0.195	<0.001
Direct LMM → overall QOL association (c′)	−0.082	−0.132 to −0.033	0.001
Indirect association through sleep quality (a×b)	−0.015	−0.025 to −0.004	0.006
Total LMM → overall QOL association (c)	−0.097	−0.148 to −0.046	<0.001

Note: The model incorporated the official 2018 CLHLS sampling weight (w_2018) together with robust standard errors. Of the 9,366 participants in the primary complete-case sample, 100 lacked an applicable official sampling weight, yielding a weighted analytic sample of 9,266. The public-use CLHLS data do not provide the geographic cluster identifiers required to reconstruct the complete multistage survey design; this analysis therefore represents a sampling-weight sensitivity analysis rather than a fully specified complex-survey analysis. The model otherwise used the same prespecified core covariates as the primary analysis. CLHLS, Chinese Longitudinal Healthy Longevity Survey; LMM, low muscle mass; QOL, quality of life; CI, confidence interval.

Table S9. Sensitivity of the indirect association to unmeasured mediator–outcome confounding.

**Residual correlation, ϱ**	**Indirect association**	**95% CI**
0.000	−0.011	−0.018 to −0.004
0.190	−0.001	−0.002 to 0.000
0.200	−0.001	−0.002 to 0.001
0.210	0.000	−0.001 to 0.001
0.220	0.001	−0.001 to 0.002
0.230	0.001	−0.000 to 0.002

Sensitivity parameters

**Parameter**	**Value**
ϱ at which the point indirect association was reduced to zero	0.210
Residual-variance R^2 product threshold	0.044
Total-variance R^2 product threshold	0.035
R^2 of the mediator model	0.053
R^2 of the outcome model	0.159

Note: Sensitivity to unmeasured mediator–outcome confounding was evaluated using the residual-correlation approach of Imai et al. The sensitivity parameter ϱ represents the correlation between the residuals of the mediator and outcome models attributable to unmeasured common causes. At $\varrho = 0$, the analysis corresponds to the no-residual-confounding assumption of the fitted linear models. The residual-variance R^2 product denotes the product of the proportions of residual variance in the mediator and outcome attributable to a hypothetical unmeasured confounder, whereas the total-variance R^2 product expresses the corresponding product relative to total variance. Estimates and confidence limits were rounded to three decimal places; statistical interpretation was based on unrounded estimates. CI, confidence interval.

Table S10. Component regression coefficients and collinearity diagnostics before and after adjustment for continuous BMI.

**Outcome**	**Predictor**	**Without BMI, B (95% CI)**	**With BMI, B (95% CI)**	**VIF with BMI**
Sleep quality	LMM	−0.066 (−0.110, −0.022)	−0.006 (−0.060, 0.048)	1.851
Overall QOL	LMM	−0.063 (−0.096, −0.029)	−0.017 (−0.058, 0.024)	1.851
Overall QOL	Sleep quality	0.163 (0.147, 0.178)	0.162 (0.146, 0.177)	1.058

Note: All models were adjusted for the prespecified core covariates. The BMI-adjusted models additionally included continuous BMI. All analyses were based on the same complete-case sample (N = 9,366). VIF values were calculated from the corresponding BMI-adjusted regression equations. These coefficients describe component associations and should not be interpreted as causal pathway effects.
